# The Book World of Medicine and Science

**Published:** 1893-03-18

**Authors:** 


### The Book World of Medicine and Science.

### REVIEWS FOR MARCH.

The North American Review contains a light article by Dr. Cyrus Edson on the "Fads of Medical Men." Among other well-known "cures," he notes "the queerest fad of modern times was that which placed the elixir of life in blue glass. That the colour of light has certain influences on life, especially life of the lower sort, as in plants, is a fact. If an animal be deprived of light it will become what is called anæmic. Reasoning from the observed effect of blue light on some plants, the inventor of the fad prescribed baths under blue glass for all. You were to strip yourself and lie down under blue glass for many hours each day. And the queerest thing about the whole business was the fact that some of the devotees not only declared themselves benefited by the treatment, but actually were so benefited."

Hypnotism is the subject of articles by Mr. Storr Best in the New Review, and by Dr. Tuckey in the *Contemporary Review*. Dr. Tuckey urges that the abuse of this powerful agent should not be brought forward as an argument against the medical employment of hypnotic suggestion, and asks what would be the mental condition of a person subjected to daily administration of chloroform for twelve months. He concludes, "if doctors take it up and apply it in suitable cases, we shall not see a method of practice which above all others demands tact and integrity handed over to unscrupulous charlatans." Mr. Storr Best is also a strong advocate, and believes "we should be able, by means of hypnotic treatment, to modify morbid processes, to arrest structural degeneration, and to awaken to more vigorous life the diseased part by improving its nutrition through an augmentation of its blood supply." We believe that there is against the habitual use of hypnotism a prejudice as strong and well-founded as against the habitual use of confession, with which it has many points in common. Its dangers can hardly be exaggerated, its benefits so far disclosed are partial and temporary even where conclusive.

Mr. Ernest Hart in the New Review has a warning to give, in view of the "Coming Cholera," against drinking between meals. "Professor Hay shows that the reaction of the empty stomach in cats is alkaline, and that considerable quantities of liquid can be swallowed without any change in ts reaction. Only solid foods can furnish the stimulus necessary for the acid secretion. Obviously under such conditions the cholera microbe might readily pass through the stomach unharmed."

Blackwood gives an entertaining picture of Aberdeen and Aberdeen doctors, apropos of Mrs. Rodger's book "Aberdeen Doctors at Home and Abroad." A good anecdote is recorded of Dr. Abernethy. "His regimen was even more ascetic than that of Sir Andrew Clark, and he put his patients into severe training on nine ounces of food per diem, with no drink, but with blue pill at discretion. A patient having confided to Sir Astley Cooper his entire belief in Dr. Abernethy's regimen, his rival cruelly said, 'I will faithfully recount to you the dinner he ate himself yesterday at the Freemasons' Tavern, where I sat next to him; he took turtle soup and punch, venison, champagne, pastry and cheese, and now he said, 'waiter, bring me a glass of brown stout.'" "We can recall an example of more disinterested and more patent inconsistency. We consulted one of the most famous Harley

THE BOOK WORLD OF MEDICINE AND SCIENCE.—*(continued)*.

Street physicians for a bad bilious attack, and were enjoined the abstinence of a St. Anthony or a Simon Stylites. We remarked that in that case we should not have the pleasure of meeting him at a gorging literary banquet which was to come off at Greenwich the next evening. 'Nonsense,' he said, 'you go there and get a seat opposite me. If you make too free with the entrées and the wines, I'll hold up my finger.'"

